# Opportunities and barriers in care for patients with post COVID-19 condition: a Delphi study among healthcare workers

**DOI:** 10.1186/s12913-026-14489-z

**Published:** 2026-04-29

**Authors:** Stella C. M. Heemskerk, Iris M. Brus, Annemieke de Groot, Lous Rijssenbeek-Nouwens, Peter Tieleman, Maike ter Wolbeek, Alex Burdorf, Sara Biere-Rafi, Juanita A. Haagsma

**Affiliations:** 1 Department of Public Health, Erasmus University Medical Center, Rotterdam, The Netherlands; 2 C-support, ‘s Hertogenbosch, The Netherlands; 3 Department of Infectious Diseases, Amsterdam University Medical Center, Amsterdam, The Netherlands

**Keywords:** Post COVID-19 condition, Long COVID, Post COVID-19 care, Long COVID care, Quality of care, Care prerequisites, Healthcare workers' perspective

## Abstract

**Background:**

Post COVID-19 condition (PCC) presents complex, long-term health challenges, yet evidence-based treatments and well-organized care pathways remain limited, creating uncertainty for healthcare professionals. This study aimed to identify healthcare workers’ (HCWs) perspectives on prerequisites, barriers and strategies for improving care for patients with PCC in the Netherlands.

**Methods:**

An online Delphi study was conducted between October 2023 and June 2024, comprising two questionnaire rounds among Dutch HCWs directly or indirectly involved in PCC care. Questionnaire themes were informed by prior research and expert input. Responses were analyzed descriptively, and feasibility ratings were assessed on a 10-point scale.

**Results:**

A total of 270 HCWs participated in round one, of whom 169 (63%) completed round two. Most participants worked in primary care and were directly involved in PCC care. One-third of HCWs reported insufficient treatment options and 25% indicated that PCC care is poorly organized. Key prerequisites included evidence-based treatment options (57%), interprofessional collaboration (34%) and scientific research (31%). Major barriers were lack of specialized PCC treatment centers (38%), limited recognition of PCC (27%) and absence of medication and treatments (25%). Broadening the professional knowledgebase and establishing specialized PCC treatment centers were ranked as top strategies to improve PCC care and were considered feasible. HCWs rated their own PCC knowledge higher than PCC knowledge of their peers (7.8 versus 5.8/10) but acknowledged gaps, particularly in treatment options and biomedical aspects. High workload and lack of capacity and time (49%), and lack of reimbursement for interprofessional consultations (43%) were major barriers for multidisciplinary collaboration.

**Conclusions:**

PCC care in the Netherlands faces significant organizational and knowledge-related challenges. Strengthening collaboration across care domains and levels, alongside targeted long-term investments in research, education, and specialized care pathways are essential to improve care quality and address identified prerequisites and barriers.

**Supplementary Information:**

The online version contains supplementary material available at 10.1186/s12913-026-14489-z.

## Background

The COVID-19 pandemic has precipitated an unprecedented global health crisis, significantly affecting not only the lives of infected individuals, but also the healthcare systems responsible for their care [1]. As the acute phase of the pandemic has receded, the need for high-quality post COVID-19 care has emerged as a critical focus for healthcare systems worldwide. Individuals who have recovered from COVID-19 often experience lingering symptoms and complications, commonly referred to as “post COVID-19 condition” (PCC) or “long COVID” [2–4]. This post-acute infectious syndrome (PAIS) presents unique challenges in terms of diagnosis, management and long-term support, necessitating new approaches to healthcare delivery.

In response to the growing prevalence and understanding of PCC, there is increasing recognition of the importance of integrated, multidisciplinary care models for recovery [5, 6]. However, healthcare professionals involved in PCC care face significant challenges including resource limitations, evolving clinical knowledge, and long-term psychological burden of providing care during the COVID-19 pandemic [7, 8]. Despite the growing body of literature on PCC, research focusing on the experiences of healthcare professionals delivering this care remains limited. This gap in the literature is critical, as understanding the perspectives of these frontline healthcare professionals is essential for identifying key opportunities, barriers, and prerequisites for delivering high-quality, patient-centred care.

Existing evidence suggests that healthcare professionals’ experiences with PCC care vary widely, influenced by factors such as organizational capacity and resource constraints, availability of care pathways and reimbursement policies [9]. Furthermore, the dynamic nature of and evolving insight in the disease itself means that healthcare professionals must continuously adapt to emerging evidence and shifting care paradigms [10]. Despite these challenges, patients and healthcare professionals have identified a number of key components necessary for effective PCC care, including improved interdisciplinary collaboration, better access to specialized care and an enhanced knowledge base [11–13]. 

Given these considerations, this study aimed to capture healthcare workers’ (HCW) experiences in delivering PCC care, with a focus on identifying prerequisites, barriers and strategies for improving care delivery. By examining these perspectives, we seek to inform future improvements in PCC care and contribute to the development of evidence-based strategies for addressing the long-term health needs of individuals affected by the pandemic.

## Methods

### Study design and context

Between October 2023 and June 2024, an online Delphi study consisting of two questionnaire rounds was conducted, targeting Dutch HCWs directly or indirectly involved in PCC care. The Delphi method is a structured approach for systematically capturing and synthesizing expert perspectives on complex or insufficiently understood topics through iterative questionnaires with anonymized feedback between rounds [14–16]. This process enables participants to reconsider their views in light of collective responses, fostering convergence toward consensus. Responses from each round were summarized and used to inform subsequent questionnaires. Given the exploratory nature of the topic and limited literature on HCW perspectives in PCC care, the Delphi technique was considered most suitable.

During the study period, both knowledge about PCC and the organization of PCC care were still evolving. A temporary reimbursement scheme for multidisciplinary primary care for PCC patients in the Netherlands was in effect from 2020 until July 2024 [17]. At the time of data collection, PCC expertise centers had not yet opened, and their structure was undefined. Centers for adults and children were established later in November 2024 and February 2025 respectively. Throughout the entire study period and up to the time of reporting, no evidence-based curative treatments for PCC were available, although options for symptom management gradually expanded.

### Panel participants

Participants were selected based on their involvement in the care for PCC patients, either directly through providing clinical care or indirectly via policy, research, or advisory roles. HCWs were invited by e-mail and through C-support’s digital communication channels. C-support is a national foundation that supports people with PCC and disseminates knowledge to healthcare professionals. Additionally, invitations were distributed via e-mail to relevant professional associations and snowball sampling was used by encouraging participants to invite colleagues from their networks to broaden outreach [18]. Participants received written online information about the study’s purpose and procedures, and provided informed consent.

### Questionnaires

The questionnaire used in this study was developed by the authors in Dutch. Questionnaire themes were informed by literature and two prior studies: (1) a Delphi study among Dutch Q-fever healthcare workers and (2) a large-scale survey among Dutch PCC patients [19–21]. A multidisciplinary advisory panel of sixteen experts, including medical specialists, general practitioners, allied health professionals, medical advisors from C-support, a data scientist and patient representatives, contributed to study design, questionnaire development and interpretation of results through four structured meetings. Additionally, an online semi-structured focus group with eleven HCWs recruited through the network of C-support, including medical specialists, general practitioners, allied health professionals and health researchers, provided further input on questionnaire themes and informed the final development of the questionnaire.

**Fig. 1:**
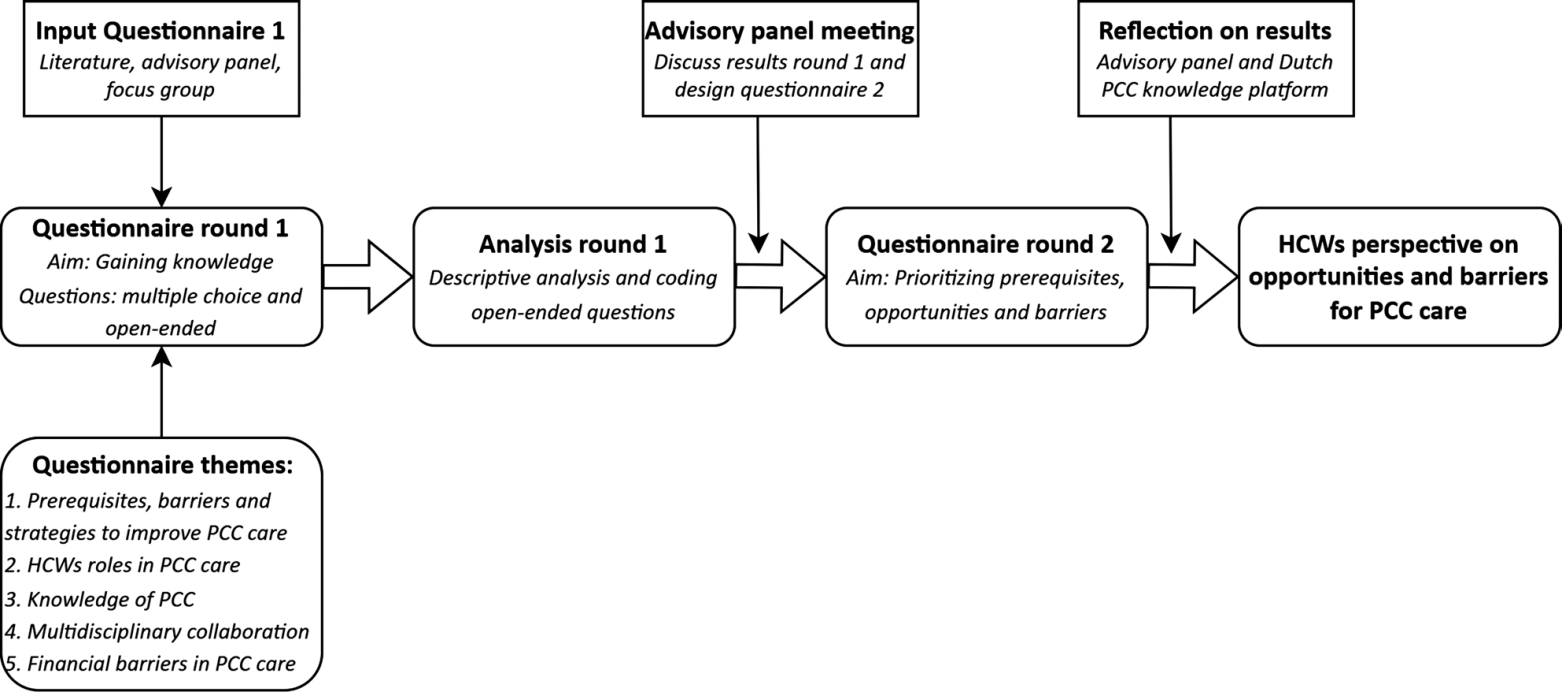
Study design and questionnaire development. HCW: healthcare worker, PCC: post COVID-19 condition

The first-round online questionnaire consisted of multiple choice questions allowing multiple responses and open-ended questions on themes such as participant characteristics and experience with PCC care, prerequisites, barriers and facilitators of PCC care, knowledge (gaps) and interdisciplinary collaboration (Fig. 1). Open-ended responses were independently coded and categorized by two researchers, using a grounded theory approach [22]. Analysis of first-round responses guided the development of the second-round questionnaire, which included more detailed items and ranking questions. Ranking items were retained for inclusion when reported by at least 5% of the participants. HCWs who completed the first-round questionnaire were invited to participate in the second round. The second-round online questionnaire primarily consisted of closed ranking questions, asking participants to prioritize a top 3 of the retained items from the first round (from least to most important). Consensus for prioritization was defined as at least 70% of participants ranking an item within their top 3. Some ranking questions were supplemented with rating questions, where participants assessed the feasibility of improvement on a scale from 0 (not feasible) to 10 (highly feasible). Ratings ≥ 6.0/10 were considered feasible.

For both rounds, responses were analyzed after two months and a maximum of three reminders. Questionnaires were available only in Dutch and could be completed exclusively online. The English translation of the second-round questionnaire is presented in Additional File 2 for informational purposes only. This study was conducted using open-source LimeSurvey software [23]. Data was pseudonymized immediately upon collection and no directly identifying data is available.

### Data analysis

For multiple choice and ranking questions, frequencies were reported. For ranking questions, mean scores and standard deviations (SD) were calculated per item. Participants selected their top three items, which were scored 3 for the highest rank, 2 for the second rank, 1 for the third rank and 0 for all remaining items. This yielded weighted mean scores ranging from 0 to 3, with higher scoring items considered more urgent and lower scoring items less urgent. For rating questions regarding feasibility of improvement and other continuous outcomes, mean (SD) scores were reported. Subgroup analyses explored differences in mean rank scores by professional groups (allied health professionals, occupational physicians, medical specialists and general practitioners). Continuous outcomes were compared using Mann–Whitney U or Kruskal–Wallis tests, categorical outcomes using Chi square tests, with *p* < 0.05 considered statistically relevant. Findings were reported only where significant and substantial deviations were observed. Data analyses were conducted using SPSS version 29 (IBM Corp., Armonk, NY, USA).

## Results

### Panel characteristics

In the first round, 270 healthcare professionals participated, of whom 169 (63%) also completed the second questionnaire. Approximately two-thirds of the panel were female, and most were aged 41 years or older. The panel represented a broad range of professions with the majority working in primary care (Table 1, Additional file 1: Table S1). Most participants (95%) were directly involved in PCC care and 52% treated or supported over five PCC patients per month. No significant sociodemographic differences were observed between participants who completed both rounds (*n* = 169) and those who only participated in the first round (*n* = 101).

**Table 1:** Characteristics of healthcare workers panel

	1st round: *n* = 270		2nd round: *n* = 169	
**Gender, ** ***n*** ** (%)**				
Female	178	65.9%	113	66.9%
Male	87	32.2%	55	32.5%
Other/prefer not to say	5	1.8%	1	0.6%
**Age (categories)**, n (*%*)				
21–30 years	30	11.1%	18	10.7%
31–40 years	43	15.9%	24	14.2%
41–50 years	58	21.5%	35	20.7%
51–60 years	79	29.3%	53	31.4%
≥ 61 years	60	22.2%	39	23.1%
**Profession (categories)**, n (*%*)^1^				
Allied health professional	112	41.5%	70	41.4%
Occupational physician or reintegration specialist	71	26.3%	44	26.0%
Medical specialist or general medical practitioner	57	21.1%	32	18.9%
General practitioner	20	7.4%	16	9.5%
Other HCW	10	3.7%	7	4.1%
**Years in profession**, median (IQR)	20	9.75–28.25	22	10.00–29.00

^1^ Additional File 1: Table S1 provides an overview of all included professions per category

HCW: healthcare worker; IQR: Interquartile range

### Current state of PCC care

According to 33% of HCWs, treatment options for PCC patients are insufficient, while 47% reported being satisfied with the care they can provide. Medical specialists (65%) and general practitioners (53%) more frequently perceived a lack of adequate treatment options compared with occupational physicians (26%) and allied health professionals (16%), and were less often satisfied (19–20% versus 37–71%). Overall, 25% of HCWs considered PCC care to be poorly organized. Medical specialists (39%) reported poor organization of PCC care more often than other HCW categories (11–25%).

HCWs considered advising on and/or offering paced activity to patients the best organized aspect of PCC care (53%), followed by providing personalized treatment (47%), multidisciplinary care (41%) and applying a biopsychosocial approach (37%) (Additional file 1: Table S2). Although multidisciplinary care was among the most frequently reported well-organized aspects, collaboration between healthcare professionals was rated relatively low, with a mean score of 5.5/10 (SD 1.8). General practitioners gave the lowest rating (mean 4.7/10), while allied health professionals rated highest (mean 5.9/10).

### Prerequisites and barriers for PCC care

HCWs identified several prerequisites for PCC care (Table 2). The most frequently mentioned were evidence-based treatment options (57%), collaboration with HCWs or within a care network (34%), scientific research (31%) and sufficient capacity, time and facilities (30%). The latter prerequisite was ranked significantly higher for medical specialists (mean score 0.88) and general practitioners (mean 0.75), compared with occupational physicians (mean 0.27) and allied health professionals (mean 0.47). Overall, improvement of adherence to the prerequisites in PCC care was considered feasible (mean scores ≥ 6.1), although organizational aspects, such as increasing capacity, time, and facilities and improving financing of care, were rated less feasible (both mean score 5.6).

**Table 2:** Ranking of prerequisites for post COVID-19 condition care

Prerequisites	Top 3^a^	Mean score, SD (0–3)^b^	Mean feasibility score (1–10)^c^
Evidence-based treatment options	57%	1.09 (1.13)	6.1
Collaboration with HCWs/within care network	34%	0.66 (1.04)	6.8
Scientific research	31%	0.72 (1.16)	6.3
Sufficient capacity, time, and facilities	30%	0.53 (0.92)	5.6
Referral by HCWs and improved referral pathways	27%	0.55 (1.02)	6.8
Financing of care	26%	0.56 (1.03)	5.6
Clear vision and policy	22%	0.47 (0.98)	6.4
Treatment guidelines/protocols	21%	0.43 (0.88)	7.0
Knowledge platforms and information provision	21%	0.40 (0.82)	7.3
Recognition, understanding, and attention for patients	17%	0.33 (0.80)	7.6

^**a**^ Percentage of HCWs who placed prerequisite in top 3

^b^ The mean score is based on weighted HCW rankings. A higher mean score indicates that a larger proportion of HCWs included the item in their top 3 and/or ranked it higher within the top 3

^c^ HCWs rated the feasibility of improving prerequisites for those prerequisites they ranked in their top 3

HCW: healthcare worker

HCWs reported several barriers to improving PCC care (Table 3). Most frequently reported were the availability and accessibility of specialized PCC treatment centers (38%), recognition and understanding of PCC (27%) and the availability of medication and (evidence-based) treatments (25%). The two most frequently reported barriers were considered feasible to resolve (mean 6.6–7.1/10). Barriers rated with the highest perceived feasibility for improvement (mean > 7.5/10) were prioritized by 10–12% of HCWs, hence regarded as less urgent, and included offering personalized treatment, facilitating knowledge exchange and creating insight into post-exertional malaise.

HCWs also identified strategies to improve PCC care. In line with the most important barriers, broadening professional knowledge, the establishment of specialized PCC treatment centers and fostering recognition and understanding for PCC patients were ranked among the top 3 by 50–56% of the HCWs (Additional file 1: Table S3). Overall, implementing these strategies was considered feasible (mean scores ≥ 6.5/10).

**Table 3:** Ranking of barriers to address in post COVID-19 condition care

Barriers	Top 3^a^	Mean score, SD (0–3)^b^	Mean feasibility score (1–10)^c^
Specialized PCC treatment centers and access to experts/specialists	38%	0.78 (1.13)	6.6
Recognition and understanding of PCC	27%	0.55 (1.05)	7.1
Availability of medication and evidence-based treatments	25%	0.46 (0.89)	5.7
Care for patients with very low tolerance for exertion	23%	0.45 (0.91)	5.9
Insight into what (does not) work for whom	23%	0.44 (0.89)	5.6
Understanding of underlying problems and mechanisms	20%	0.38 (0.87)	6.1
Multidisciplinary approach to care	15%	0.27 (0.71)	7.3
Personalized treatment	12%	0.22 (0.66)	8.0
Knowledge exchange among professionals through a national network	12%	0.22 (0.66)	7.7
Diagnostic methods and accurate diagnosis	11%	0.22 (0.71)	5.3
Approach based on the biopsychosocial model	11%	0.22 (0.70)	7.1
Clear coordination of care	10%	0.22 (0.72)	5.9
Insight into post-exertional malaise (PEM)	10%	0.20 (0.66)	7.6
Rapid implementation of new insights	9%	0.17 (0.61)	7.5
Adequate knowledge among HCWs	8%	0.20 (0.68)	7.4
Accessibility of care	8%	0.15 (0.57)	5.8
Sufficient treatment options	8%	0.14 (0.51)	6.5
Insight into subtypes of PCC	6%	0.12 (0.51)	6.9
Clear PCC definition	6%	0.09 (0.41)	6.0
PCC specific guidance and support	5%	0.12 (0.55)	6.9
Structural embedding of care services	4%	0.09 (0.47)	6.0

^a^ Percentage of HCWs who placed barrier in top 3

^b^ The mean score is based on weighted HCW rankings. A higher mean score indicates that a larger proportion of HCWs included the item in their top 3 and/or ranked it higher within the top 3

^c^ HCWs rated the feasibility of addressing barriers for those barriers they ranked in their top 3

HCW: healthcare worker; PCC: post COVID-19 condition; PEM: post-exertional malaise

### Healthcare workers’ roles

According to HCWs, optimal PCC care requires the involvement of occupational therapists (78%), general practitioners (53%), physiotherapists (49%), occupational physicians (33%) and rehabilitation physicians (28%). For PCC diagnosis, the general practitioner was considered the most suitable professional (89%), followed by the rehabilitation physician (55%) and internal medicine physician (30%). The general practitioner was also viewed as the most appropriate primary treating physician (81%), followed by the rehabilitation physician (62%) and occupational therapist (33%). Several respondents emphasized the need for a primary care coordinator to coordinate collaboration between all involved parties, monitor treatment options and progress, and guide patients through complex care pathways. For this coordinating role, the general practitioner (63%), occupational physician (39%) and rehabilitation physician (36%) were deemed most suitable.

### Knowledge gaps and improvement strategies

Participants rated their own PCC knowledge higher than that of their peers (mean 7.8/10 [SD 1.1] vs. 5.8/10 [SD 1.3]) and recognized both personal knowledge gaps (75%) and knowledge gaps among peers (91%). General practitioners rated their overall PCC knowledge significantly lower (mean 7.0/10) compared with medical specialists (mean 8.1/10) and allied health professionals (mean 7.8/10).

The most reported knowledge gaps concerned treatment options (63%), biomedical aspects (51%) and exertional intolerance, PEM and postural orthostatic tachycardia syndrome (37%) (Table 4). The knowledge gap concerning treatment options was ranked significantly lower by allied health professionals (mean score 0.93) compared with general practitioners (mean 1.75) and medical specialists (1.63). Closing the knowledge gaps was considered feasible (mean scores 6.2–7.2/10). The most commonly suggested strategies to improve knowledge were sharing emerging scientific insights (50%), establishing specialized PCC centers (42%) or developing and revising treatment guidelines/protocols in line with emerging scientific evidence (41%) (Additional file 1: Table S4).

**Table 4:** Ranking of knowledge gaps in post COVID-19 condition care

Knowledge gaps	Top 3^a^	Mean score, SD (0–3)^b^	Mean feasibility score (1–10)^c^
Knowledge of treatment options (including medication and follow-up care)	63%	1.19 (1.13)	6.8
Biomedical knowledge gaps (including underlying mechanisms)	51%	1.06 (1.23)	6.2
Knowledge of exertional tolerance, PEM, and POTS	37%	0.73 (1.07)	7.2
Knowledge of long-term consequences, recovery, and prognosis of PCC	35%	0.70 (1.04)	7.1
Awareness of ongoing studies and research findings	30%	0.59 (1.02)	7.2
Knowledge of environmental and psychosocial factors influencing recovery (including lifestyle)	24%	0.54 (1.04)	6.0
Keeping knowledge and guidelines up to date	23%	0.42 (0.86)	6.4
Knowledge of referral options and experience of other HCWs	17%	0.29 (0.69)	6.6
Knowledge of diagnostic methods	13%	0.27 (0.77)	6.9
Knowledge of PCC symptoms	10%	0.22 (0.72)	6.3

^**a**^ Percentage of HCWs who placed knowledge gap in top 3

^b^ The mean score is based on weighted HCW rankings. A higher mean score indicates that a larger proportion of HCWs included the item in their top 3 and/or ranked it higher within the top 3

^c^ HCWs rated the feasibility of addressing the knowledge gaps for those gaps they ranked in their top 3

HCW: healthcare worker; PCC: post COVID-19 condition; PEM: Post-exertional malaise; POTS: Postural orthostatic tachycardia syndrome

### Multidisciplinary collaboration

The most frequently reported barriers to multidisciplinary collaboration included insufficient collaboration among healthcare professionals (50%), high workload and insufficient capacity and time (49%), and lack of reimbursement for interprofessional consultations (43%) (Table 5). The prioritization of the latter barrier was significantly higher for allied health professionals (mean score 1.56), compared with other professional groups (mean scores 0.14–0.69). General practitioners (mean 1.25) and medical specialists (mean 1.03) ranked a lack of knowledge among healthcare professionals significantly higher than allied health professionals (mean 0.53) and occupational physicians (mean 0.41), while occupational physicians prioritized the lack of low-threshold contact between healthcare professionals higher (mean 1.09) than other professional groups (mean range 0.13–0.44). Addressing workload, capacity and time, as well as reimbursement for interprofessional contact was considered poorly feasible (mean 4.4/10 and 5.7/10, respectively). Key priorities for future collaboration include strengthening the linkages between the work, healthcare, and social domains (30%) and ensuring that care is provided in the most appropriate setting (27%).

**Table 5:** Ranking of barriers for multidisciplinary collaboration in post COVID-19 condition care

Multidisciplinary collaboration barriers	Top 3^a^	Mean score, SD (0–3)^b^	Mean feasibility score (1–10)^c^
Lack of (multidisciplinary) collaboration between healthcare professionals	50%	0.89 (1.07)	6.6
High workload and lack of capacity/time	49%	1.14 (1.27)	4.4
Lack of reimbursement for interprofessional consultations	43%	0.86 (1.15)	5.7
Unfamiliarity with collaboration opportunities/added value of other disciplines	39%	0.76 (1.08)	6.8
Lack of knowledge among healthcare professionals	37%	0.70 (1.08)	7.2
Differences in vision regarding treatment and guidance	36%	0.71 (1.05)	5.6
Poor information transfer (e.g., one-way communication, lack of feedback and reporting)	24%	0.47 (0.92)	6.8
No low-threshold contact/short communication lines between healthcare professionals	23%	0.47 (0.92)	6.6

^a^ Percentage of HCWs who placed barrier in top 3

^b^ The mean score is based on weighted HCW rankings. A higher mean score indicates that a larger proportion of HCWs included the item in their top 3 and/or ranked it higher within the top 3

^c^ HCWs rated the feasibility of addressing the barrier for those barriers they ranked in their top 3

## Discussion

This study showed that the organization and availability of care for PCC patients leaves considerable room for improvement. While nearly half of HCWs were satisfied with the care they provide, a substantial proportion reported insufficient treatment options as well as poor organization of PCC care not adequately meeting patients’ needs. HCWs identified several prerequisites for high-quality care, most notably availability, and hence development, of evidence-based treatment options, formalized and coordinated multidisciplinary collaboration, and continued scientific research to address knowledge gaps. The most frequently reported barriers were limited access to specialized PCC centers and lack of recognition and understanding of PCC, both considered feasible to address. Strategies such as establishing specialized treatment centers and enhancing professional knowledge were ranked as top priorities and viewed as achievable. These findings underscore the perceived need for structural improvements in PCC care, yet, rankings varied widely, and no single factor consistently emerged as a top priority. For rankings on prerequisites, barriers and strategies to improve PCC care, the highest-ranked items were placed in the top 3 by only 38 to 57% of participants, with relatively low mean scores, indicating limited consensus among HCWs. The absence of any item meeting the 70% consensus threshold highlights limited agreement among HCWs on where improvements should be directed.

Overall, the prioritized aspects per theme appeared largely consistent across professional groups, although some prerequisites and multidisciplinary collaboration barriers for PCC care differed between medical professionals and allied health professionals. Medical professionals prioritized evidence-based treatment options and sufficient capacity, time and facilities more strongly than allied health professionals, likely reflecting their diagnostic role and the current lack of evidence-based medical treatments. Allied health professionals and occupational physicians, who mainly focus on functional rehabilitation, appeared to prioritize different aspects of PCC care. These differences likely reflect variation in their roles, daily practice, and the resources and constraints within their respective care settings. In particular, their priorities concerned practical and organizational aspects of care delivery.

Our findings among HCWs align with previous research from the patient perspective. PCC patients often report uncertainty about where to seek help, limited access to healthcare professionals with appropriate knowledge and skills, and difficulties obtaining support with diagnosis and symptom management. [21, 24, 25] Furthermore, PCC patients, like other PAIS patients [26, 27], frequently experience a lack of recognition from healthcare professionals. [24, 28] However, recognition, as an integral component of person-centered care, is considered crucial for facilitating integrated primary care from the perspective of individuals living with chronic conditions and multiple care needs [29]. Extending beyond the patient perspective, our findings also resonate with previous research on Q-fever care, where similar challenges were identified, including knowledge gaps, limited recognition and the need for clearer guidance and structured collaboration [19]. While these challenges are not unique to PCC care, they appear more prominent in the context of PCC due to the large patient population, the rapid emergence of the condition and the continuously evolving evidence base, which together place additional demands on healthcare organization and coordination.

While participants indicated that the general practitioner, as gatekeeper to secondary care, should play a central role in PCC diagnosis and act as the primary treating physician and coordinator, the complexity of PCC, characterized by multiple symptoms across organ systems, necessitates an integrated, cross-disciplinary approach. According to HCWs, multidisciplinary care is essential within this framework and this aligns with the patient perspective showing that patients experience problems across multiple domains and consult professionals from different disciplines [19]. This need is also emphasized in literature and the Dutch PCC guideline, which advises healthcare professionals to consider multidisciplinary treatment in primary care, ensure alignment of interventions, and refer to secondary care in cases of high complexity [30–33]. Despite this, collaboration was rated poorly (mean 5.5/10), even though collaboration with HCWs or within a care network was ranked as the second-highest prerequisite for PCC care. Notably, general practitioners rated collaboration between HCWs lowest despite being considered to play a central role in PCC care. This is consistent with previous observations in a Dutch setting, where insufficient organization of multidisciplinary care limits opportunities for general practitioners to engage in collaboration [31]. A potential explanation for insufficient collaboration is the lack of clearly defined roles and responsibilities among healthcare professionals, which may hinder high-quality care and patient outcomes [34–36]. 

### From research to practice

The large number and diversity of prerequisites and barriers identified for PCC care reflect its complexity, with no clear consensus on the most urgent needs or most effective solutions. This suggests that no single, ready-made solution will be sufficient to improve quality of care. Instead, our findings highlight the need for structured PCC care pathways to improve patient outcomes. Such pathways should be patient-centered and integrated, grounded in acknowledging patients’ experiences and needs. Establishing these pathways requires adequate professional education, as well as clear roles and guidance for HCWs. In addition, there is substantial room to strengthen collaboration across care domains, particularly between primary and secondary care providers, and between occupational physicians, curative and specialist care. Improving awareness of collaboration opportunities and the added value of different disciplines may facilitate more effective integration of care. Limited recognition of professionals’ roles within PCC care may further hinder patient access to appropriate healthcare services [37]. 

Promising steps toward integrated care and knowledge development have been taken in the Netherlands after the study period. PCC expertise centers were opened with the aim to deliver high-quality PCC care to a select group of patients and to accelerate knowledge development and dissemination, and the national Post-COVID Network Netherlands was established to align research and care. However, without structural financing, continuity and long-term sustainability of these initiatives to support responsive, evidence-based PCC care remains uncertain. Moreover, rapid developments in PCC research and clinical practice require timely guideline updates and pragmatic, patient-centered approaches to ensure that care remains responsive to patients’ needs. Conventional evidence standards and procedural frameworks may be too time-consuming to provide timely guidance. PCC expertise centers could serve as platforms for knowledge dissemination, practice-based learning and research, but sustained structural funding is essential for its realization. Furthermore, the responsibility for designing and implementing programs to enhance PCC care lies with policymakers, while HCWs play a crucial role in identifying gaps and improving care.

### Strengths and limitations

This study is among the first to systematically explore HCWs perspectives on PCC care in the Netherlands, addressing an urgent and evolving healthcare challenge. The use of the Delphi method allowed for structured consensus-building among a diverse panel of experts and capturing informed opinions on a complex topic with limited evidence. The multidisciplinary composition of the panel ensured comprehensive perspectives. Furthermore, the study achieved strong engagement, with 270 participants in the first round and 169 completing both rounds, representing a broad range of care settings.

Despite these strengths, some limitations should be noted. First, questionnaire themes were intentionally defined broadly due to the exploratory design of the study and the scarcity of prior evidence, which may have limited the degree of consensus achieved. While the findings provide a valuable first step toward improving PCC care in the Netherlands, more in-depth research, such as qualitative interviews is required to translate these insights into actionable recommendations. Second, only two Delphi rounds were conducted. While this aligns with common practice, additional rounds might have improved assessment of response stability and ranking consistency [38, 39]. Further iteration in a third round was unlikely to provide additional insights or consensus because of the large number and heterogeneity of identified challenges, in line with the exploratory nature of the study, as well as feasibility considerations. However, with only two rounds, response stability could not be fully assessed and additional rounds might have led to further convergence. Therefore, conclusions regarding prioritization should be interpreted with caution. Third, the recruitment strategy may have introduced selection and non-response bias, potentially overrepresenting HCWs with a particular interest in PCC. Allied health professionals and occupational physicians were more strongly represented in the panel than general practitioners and medical specialists, which may have influenced the prioritization and ratings. Subgroup analyses were conducted to explore this imbalance and indicated some priorities differed by professional groups. As such, representativeness across all professional groups may be limited. Nonetheless, the inclusion of a diverse range of stakeholders enhances the overall relevance and applicability of the findings. Fourth, the study was conducted during a period of rapid developments in PCC care, meaning some findings may have been influenced by evolving policies and resources. Finally, findings reflect the Dutch healthcare context and may not be fully generalizable. However, the key challenges that were identified, such as lack of evidence-based treatment options and knowledge gaps, are common across healthcare systems, making these insight broadly relevant at an international level.

## Conclusions

This Delphi study identified significant gaps in treatment options, care organization, and professional knowledge for PCC in the Netherlands. Strengthening collaboration between primary and secondary care alongside the establishment of specialized PCC centers and the development of updated guidelines, emerged as the highest ranked strategies to improve care. However, the limited consensus in rankings indicates that perceived priorities are not uniformly shared and that feasibility should be interpreted with caution. Targeted long-term investments in research, care pathways and infrastructure, and education remain essential to address these challenges and support progress toward high-quality, coordinated PCC care.

## Supplementary Information

Below is the link to the electronic supplementary material.

Supplementary Material 1

Supplementary Material 2

## Data Availability

The dataset generated and analyzed during the current study is available from the corresponding author on reasonable request.

## Abbreviations

HCW: Healthcare worker
IQR: Interquartile range
PAIS: Post-acute infectious syndrome
PCC: Post COVID-19 condition
PEM: Post-exertional malaise
POTS: Postural orthostatic tachycardia syndrome
SD: Standard Deviation
