# Supplementary material for: Opportunities and barriers in care for patients with post COVID-19 condition: a Delphi study among healthcare workers
Source: BMC Health Serv Res. 2026 Apr 29;26:835. doi: 10.1186/s12913-026-14489-z (PMC13270559; doi:10.1186/s12913-026-14489-z)
Supplement: Supplementary file 1 — Supplementary Material 1 [file 12913_2026_14489_MOESM1_ESM.docx]

**Additional File 1: Opportunities and barriers in care for patients with post COVID-19 condition: A Delphi study among healthcare workers**

**Table S1. Professions and corresponding categories of healthcare workers panel**

|  | **1^st^ round:**  **n=270** | | **2^nd^ round:**  **n=169** | |
| --- | --- | --- | --- | --- |
| **Allied health professional***,* n (*%*) | **112** | **41.5%** | **70** | **41.4%** |
| Occupational therapist | 44 | 16.3% | 31 | 18.3% |
| Physiotherapist | 34 | 12.6% | 19 | 11.2% |
| Hospital nurse | 6 | 2.2% | 2 | 1.2% |
| Dietitian or nutritionist | 5 | 1.9% | 5 | 3.0% |
| Psychologist or psychotherapist | 5 | 1.9% | 2 | 1.2% |
| Nurse (non-hospital) | 4 | 1.5% | 2 | 1.2% |
| Social worker | 4 | 1.5% | 3 | 1.8% |
| Nurse practitioner | 3 | 1.1% | 3 | 1.8% |
| Medical assistant | 2 | 0.7% | 0 | 0.0% |
| Speech therapist | 2 | 0.7% | 1 | 0.6% |
| Home care nurse | 1 | 0.4% | 1 | 0.4% |
| Manual therapist | 1 | 0.4% | 1 | 0.6% |
| Practice Nurse (somatic care within GP practice) | 1 | 0.4% | 0 | 0.0% |
| **Occupational physician or reintegration specialist,** n (*%*) | **71** | **26.3%** | **44** | **26.0%** |
| Occupational physician | 66 | 24.4% | 43 | 25.4% |
| Reintegration coordinator | 4 | 1.5% | 1 | 0.6% |
| Occupational health expert | 1 | 0.4% | 0 | 0.0% |
| **Medical specialist or general medical practitioner,** n (*%*) | **57** | **21.1%** | **32** | **18.9%** |
| Rehabilitation physician | 14 | 5.2% | 8 | 4.7% |
| Pulmonologist | 11 | 4.1% | 7 | 4.1% |
| Sports physician | 9 | 3.3% | 5 | 3.0% |
| General medical practitioner (not specialized) | 6 | 2.2% | 5 | 3.0% |
| Psychiatrist | 6 | 2.2% | 3 | 1.8% |
| Pediatrician | 3 | 1.1% | 2 | 1.2% |
| Internal medicine physician | 2 | 0.7% | 0 | 0.0% |
| Cardiologist | 1 | 0.40% | 0 | 0.00% |
| Gastroenterologist | 1 | 0.40% | 1 | 0.60% |
| Insurance physician | 1 | 0.40% | 0 | 0.00% |
| Public health physician | 1 | 0.40% | 0 | 0.00% |
| Rheumatologist | 1 | 0.40% | 1 | 0.60% |
| Youth health physician | 1 | 0.40% | 0 | 0.00% |
| **General practitioner,** n (*%*) | **20** | **7.4%** | **16** | **9.5%** |
| **Other healthcare worker,** n (*%*) | **10** | **3.7%** | **7** | **4.1%** |
| Executive/Manager | 3 | 1.1% | 2 | 1.2% |
| Acupuncturist | 1 | 0.40% | 1 | 0.60% |
| Body stress release practitioner | 1 | 0.40% | 0 | 0.00% |
| Body-oriented therapist | 1 | 0.40% | 1 | 0.60% |
| Breathing and relaxation therapist | 1 | 0.40% | 0 | 0.00% |
| Health insurance employee | 1 | 0.40% | 1 | 0.60% |
| Policy maker | 1 | 0.40% | 1 | 0.60% |
| Sepsis educator | 1 | 0.40% | 1 | 0.60% |

GP: general practitioner

**Table S2. Ranking of best organized aspects of post COVID-19 condition care**

| **Best organized aspects** | **Top 3^a^** | **Mean score, SD**  **(0-3)^b^** |
| --- | --- | --- |
| Advising on activity pacing | 53% | 1.05 (1.15) |
| Personalized treatment | 47% | 0.92 (1.15) |
| Multidisciplinary approach to care | 41% | 0.78 (1.04) |
| Approach based on the biopsychosocial model | 37% | 0.78 (1.16) |
| Estimating activity and rest | 34% | 0.67 (1.04) |
| Insight into post-exertional malaise (PEM) | 30% | 0.59 (1.01) |
| PCC specific guidance and support | 16% | 0.30 (0.78) |
| Clear PCC definition | 7% | 0.21 (0.75) |
| Assessment of mental load capacity | 9% | 0.16 (0.56) |
| Recognition of PCC | 6% | 0.13 (0.56) |
| Assessment of treatment goals | 7% | 0.12 (0.51) |

^a^ Percentage of HCWs who placed aspect in top 3.
^b^ The mean score is based on weighted HCW rankings. A higher mean score indicates that a larger proportion of HCWs included the item in their top 3 and/or ranked it higher within the top 3.
HCW: healthcare worker; PCC: post COVID-19 condition; PEM: post-exertional malaise

**Table S3. Ranking of strategies to improve post COVID-19 condition care**

| **Improvement strategies** | **Top 3^a^** | **Mean score, SD (0-3)^b^** | **Mean feasibility score (1-10)^c^** |
| --- | --- | --- | --- |
| Knowledge expansion (through training, continuing education, scientific research) | 57% | 1.14 (1.16) | 6.9 |
| Specialized PCC treatment centers and specialized care | 56% | 1.20 (1.24) | 6.5 |
| Understanding, recognition, and attention for the patient | 50% | 1.02 (1.21) | 7.7 |
| Clear care pathway and political policy | 44% | 0.94 (1.18) | 6.0 |
| Financing of care | 37% | 0.61 (0.93) | 6.0 |
| Improving communication with and between healthcare providers (communication and information platform) | 30% | 0.57 (0.96) | 6.9 |
| Implementing care coordinators | 13% | 0.24 (0.67) | 6.9 |

^a^ Percentage of HCWs who placed strategy in top 3.
^b^ The mean score is based on weighted HCW rankings. A higher mean score indicates that a larger proportion of HCWs included the item in their top 3 and/or ranked it higher within the top 3.
^c^ HCWs rated the feasibility of implementing strategies for those strategies they ranked in their top 3.
HCW: healthcare worker; PCC: post COVID-19 condition

**Table S4. Ranking of strategies to improve healthcare workers’ knowledge on post COVID-19 condition**

| **Improvement strategies (knowledge)** | **Top 3^a^** | **Mean score, SD**  **(0-3)^b^** |
| --- | --- | --- |
| Sharing advancing scientific knowledge | 50% | 0.93 (1.11) |
| Developing, revising, and keeping multidisciplinary guidelines and recommendations up to date | 41% | 0.92 (1.22) |
| Establishing specialized PCC centers | 42% | 0.87 (1.17) |
| Improving (multidisciplinary) collaboration between HCWs | 34% | 0.62 (0.93) |
| More (scientific) research | 25% | 0.53 (1.01) |
| Raising awareness of (the severity of) PCC (e.g. professional literature, training, media) | 21% | 0.49 (1.01) |
| Implementing an integrated approach | 26% | 0.47 (0.90) |
| Gaining and sharing knowledge and experience | 21% | 0.44 (0.95) |
| Education and continuing training | 23% | 0.42 (0.86) |
| Improving information provision (information platform) | 17% | 0.30 (0.73) |

^a^ Percentage of HCWs who placed strategy in top 3.
^b^ The mean score is based on weighted HCW rankings. A higher mean score indicates that a larger proportion of HCWs included the item in their top 3 and/or ranked it higher within the top 3.
HCW: healthcare worker; PCC: post COVID-19 condition
