# Supplementary material for: Opportunities and barriers in care for patients with post COVID-19 condition: a Delphi study among healthcare workers
Source: BMC Health Serv Res. 2026 Apr 29;26:835. doi: 10.1186/s12913-026-14489-z (PMC13270559; doi:10.1186/s12913-026-14489-z)
Supplement: Supplementary file 2 — Supplementary Material 2 [file 12913_2026_14489_MOESM2_ESM.docx]

**Additional File 2: Opportunities and barriers in care for patients with post COVID-19 condition: A Delphi study among healthcare workers**

**Delphi Study post-COVID-19 condition care: second-round questionnaire***

* This additional file presents an English translation of the original Dutch questionnaire send out to the participants in the second Delphi-round (based on AI-generation). This translation is non-official and does not meet formal research translation standards; it is provided for informational purposes only.

Abbreviations: HCW: healthcare worker; PCC: post-COVID-19 condition

**Table of contents**

| **Module 1** | Prerequisites, barriers and strategies to improve PCC care |
| --- | --- |
| **Module 2** | Healthcare workers’ roles in PCC care |
| **Module 3** | Multidisciplinary collaboration in PCC care |
| **Module 4** | Financial barriers in PCC care |
| **Module 5** | Knowledge of PCC |

**Module 1. Prerequisites, barriers and strategies to improve PCC care**

The following questions concern the success factors, areas for improvement, and requirements in the current care offer for PCC patients.

1. **Have you treated or supported PCC patients?**
   □ Yes
   □ No

**Best organized aspects of PCC care**
In the first round of this study, participants were asked what is going well in the care for PCC patients. For 48%, this is pacing activities; for 44%, the approach based on the biopsychosocial model; for 28%, offering personalized treatment; for 27%, assessing activities and rest; and for 24%, access to/availability of multidisciplinary care.

1. **According to you, what are the most important success factors in the current care for PCC patients?**
   Double-click or click-and-drag the three most important success factors from the list on the right to the list on the left. Arrange the items in the left list in order of importance, placing the most important item at the top.

| - **Approach based on the biopsychosocial model** |
| --- |
| - **Advising on activity pacing** |
| - **Clear PCC definition** |
| - **Personalized treatment** |
| - **Estimating activity and rest** |
| - **Assessment of treatment goals** |
| - **Assessment of mental load capacity** |
| - **Insight into post-exertional malaise (PEM)** |
| - **PCC specific guidance and support** |
| - **Multidisciplinary approach to care** |
| - **Recognition of PCC** |

**Barriers and strategies to improve PCC care**

In addition to the best organized aspects, there are also aspects that could be improved in the care for PCC patients. In the first round of this study, 29% of participants indicated the availability of medications and evidence-based treatments, 24% mentioned multidisciplinary care, 23% highlighted care for patients with very low tolerance and the knowledge of healthcare professionals, and 22% pointed to knowledge sharing among professionals through a national network.

1. **According to you, what could be improved in the care for patients with PCC?**
   Double-click or click-and-drag the three most important aspects that could be improved from the list on the right to the list on the left. Arrange the items in the left list in order of importance, placing the most important item at the top.

| - **Recognition and understanding of PCC** |
| --- |
| - **Approach based on the biopsychosocial model** |
| - **Diagnostic methods and accurate diagnosis** |
| - **Clear PCC definition** |
| - **Specialized PCC treatment centers and access to experts/specialists** |
| - **Availability of medication and evidence-based treatments** |
| - **Personalized treatment** |
| - **Sufficient treatment options** |
| - **Understanding of underlying problems and mechanisms** |
| - **Insight into post-exertional malaise (PEM)** |
| - **Insight into subtypes of PCC** |
| - **Insight into what (does not) work for whom** |
| - **Adequate knowledge among HCWs** |
| - **Knowledge exchange among professionals through a national network** |
| - **PCC specific guidance and support** |
| - **Multidisciplinary approach to care** |
| - **Clear coordination of care** |
| - **Rapid implementation of new insights** |
| - **Accessibility of care** |
| - **Structural embedding of care services** |
| - **Care for patients with very low tolerance for exertion** |

1. **To what extent do you think this improvement can be achieved?** [Only for three selected answer options from question 3]

| 1  Not at all easy to achieve | 2 | 3 | 4 | 5 | 6 | 7 | 8 | 9 | 10  Very easy to achieve |
| --- | --- | --- | --- | --- | --- | --- | --- | --- | --- |
| ❑ | ❑ | ❑ | ❑ | ❑ | ❑ | ❑ | ❑ | ❑ | ❑ |

1. **According to you, what are the best ways to improve the current care for PCC patients?**

Double-click or click-and-drag the three best ways from the list on the right to the list on the left. Arrange the items in the left list in order of importance, placing the most important item at the top.

| - **Understanding, recognition, and attention for the patient** |
| --- |
| - **Clear care pathway and political policy** |
| - **Specialized PCC treatment centers and specialized care** |
| - **Financing of care** |
| - **Implementing care coordinators** |
| - **Knowledge expansion (through training, continuing education, scientific research)** |
| - **Improving communication with and between healthcare providers (communication and information platform)** |

1. **To what extent do you think this strategy can be achieved?** [Only for three selected answer options from question 5]

| 1  Not at all easy to achieve | 2 | 3 | 4 | 5 | 6 | 7 | 8 | 9 | 10  Very easy to achieve |
| --- | --- | --- | --- | --- | --- | --- | --- | --- | --- |
| ❑ | ❑ | ❑ | ❑ | ❑ | ❑ | ❑ | ❑ | ❑ | ❑ |

**Prerequisites of PCC care**

In the first round of this study, 48% of participants indicated that they had (completely) sufficient possibilities to provide good treatment to PCC patients. For 33% of participants, this was (completely) insufficient.

1. **What do you need to provide high quality care and support to PCC patients?**

Double-click or click-and-drag the three most important requirements from the list on the right to the list on the left. Arrange the items in the left list in order of importance, placing the most important item at the top.

| - **Evidence-based treatment options** |
| --- |
| - **Sufficient capacity, time, and facilities** |
| - **Referral by HCWs and improved referral pathways** |
| - **Clear vision and policy** |
| - **Recognition, understanding, and attention for patients** |
| - **Financing of care** |
| - **Knowledge platforms and information provision** |
| - **Treatment guidelines/protocols** |
| - **Collaboration with HCWs/within care network** |
| - **Scientific research** |

1. **To what extent do you think this prerequisite can be achieved?** [Only for three selected answer options from question 7]

| 1  Not at all easy to achieve | 2 | 3 | 4 | 5 | 6 | 7 | 8 | 9 | 10  Very easy to achieve |
| --- | --- | --- | --- | --- | --- | --- | --- | --- | --- |
| ❑ | ❑ | ❑ | ❑ | ❑ | ❑ | ❑ | ❑ | ❑ | ❑ |

**Module 2. Healthcare workers’ roles in PCC care**

The following questions concern how care PCC patients should ideally look and which healthcare providers should play the most important role.

**Healthcare providers involved in care, support, and treatment of PCC patients**
In the first round of this study, 72% of participants indicated that the occupational therapist plays the most important role in care, support, and treatment for PCC patients in their region. This was followed by 66% choosing the physiotherapist, 59% the general practitioner, 26% the occupational health physician, and 22% the psychologist or psychotherapist.

1. **According to your experience, who plays the most important role in the care, support, and treatment of PCC patients?**
   Double-click or click-and-drag the three most important healthcare providers from the list on the right to the list on the left. Arrange the items in the left list in order of importance, placing the most important item at the top.

| - **Occupational physician** |
| --- |
| - **Dietitian or nutritionist** |
| - **Occupational therapist** |
| - **Physiotherapist** |
| - **General practitioner** |
| - **Internal medicine physician** |
| - **Speech therapist** |
| - **Pulmonologist** |
| - **Practice Nurse (mental health care within GP practice)** |
| - **Practice Nurse (somatic care within GP practice)** |
| - **Psychologist or psychotherapist** |
| - **Rehabilitation physician** |
| - **Sports physician** |

**Diagnosing PCC**In the first round of this study, 87% of participants indicated that the general practitioner plays the most important role in diagnosing PCC in their region. This was followed by 32% choosing the rehabilitation physician, 28% the occupational physician, 23% the physiotherapist, and 20% the occupational therapist.

1. **According to your experience, who plays the most important role in diagnosing PCC?**Only one answer is possible.

| - **Occupational physician** |
| --- |
| - **Occupational therapist** |
| - **Physiotherapist** |
| - **General practitioner** |
| - **Internal medicine physician** |
| - **Neurologist** |
| - **Rehabilitation physician** |
| - **Sports physician** |

1. **If, according to your experience, another healthcare provider also plays an important role in diagnosing PCC, who is this?**
   A maximum of two answers is possible. [Exclude the answer option from question 10]

| - There is no other healthcare provider who plays an important role in diagnosing PCC [exclusive answer] |
| --- |
| - **Occupational physician** |
| - **Occupational therapist** |
| - **Physiotherapist** |
| - **General practitioner** |
| - **Internal medicine physician** |
| - **Neurologist** |
| - **Rehabilitation physician** |
| - **Sports physician** |

**Primary treating physician**In the first round of this study, 59% of participants indicated that the general practitioner is best suited to fulfill the role of primary treating physician. This was followed by 33% choosing the rehabilitation physician, 18% the occupational therapist, 12% the physiotherapist, and 8% the sports physician.

1. **Who do you think is best suited to fulfill the role of primary treating physician?**
   Only one answer is possible. A main treating physician is considered the person who has an overall view of the treatment and ensures that everything related to the treatment is well coordinated.

| - **Occupational physician** |
| --- |
| - **Occupational therapist** |
| - **Physiotherapist** |
| - **General practitioner** |
| - **Internal medicine physician** |
| - **Pulmonologist** |
| - **Rehabilitation physician** |
| - **Sports physician** |

1. **If, according to your experience, another healthcare provider can also fulfill the role of primary treating physician, who is this?**

A maximum of two answers is possible. [Exclude the answer option from question 12]

| - There is no other healthcare provider who can also fulfill the role of primary treating physician [exclusive answer] |
| --- |
| - **Occupational physician** |
| - **Occupational therapist** |
| - **Physiotherapist** |
| - **General practitioner** |
| - **Internal medicine physician** |
| - **Pulmonologist** |
| - **Rehabilitation physician** |
| - **Sports physician** |

**Primary care coordinator**In the first round of this study, 56% of participants indicated that the general practitioner is best suited to fulfill the role of primary care coordinator. This was followed by 27% choosing the rehabilitation physician, 22% the practice nurse (somatic care), 21% the occupational therapist, and 17% the practice nurse (mental health care).

1. **Who do you think is best suited to fulfill the role of primary care coordinator?**
   Only one answer is possible. A primary care coordinator is considered the person who ensures coordination and management of collaboration between involved parties and monitors the progress of care and support.

| - **Occupational physician** |
| --- |
| - **Occupational therapist** |
| - **Physiotherapist** |
| - **General practitioner** |
| - **Pulmonologist** |
| - Social worker |
| - **Practice Nurse (mental health care within GP practice)** |
| - **Practice Nurse (somatic care within GP practice)** |
| - **Rehabilitation physician** |
| - **Nurse** |

1. **If, according to your experience, another healthcare provider can also fulfill the role of primary care coordinator, who is this?**
   A maximum of two answers is possible. [Exclude the answer option from question 14]

| - **There is no other healthcare provider who can also fulfill the role of primary care coordinator [exclusive answer]** |
| --- |
| - **Occupational physician** |
| - **Occupational therapist** |
| - **Physiotherapist** |
| - **General practitioner** |
| - **Pulmonologist** |
| - Social worker |
| - **Practice Nurse (mental health care within GP practice)** |
| - **Practice Nurse (somatic care within GP practice)** |
| - **Rehabilitation physician** |
| - **Nurse** |

1. **According to you, what are the most important tasks of a primary care coordinator?**
   Double-click or click-and-drag the three most important tasks from the list on the right to the list on the left. Arrange the items in the left list in order of importance, placing the most important item at the top.

| - Attention to and personal contact with the patient and advocacy |
| --- |
| - Point of contact for patient and healthcare provider |
| - Aligning and coordinating care |
| - Guiding and supporting the process |
| - Facilitating communication between all parties involved |
| - Facilitating collaboration |
| - Informing the patient |
| - Identifying the care needs and treatment options |
| - Knowledge about PCC and treatment options |

**Module 3. Multidisciplinary collaboration in PCC care**

**Success factors**In the first round of this study, 43% of participants indicated that certain aspects of collaboration in PCC care are well organized. The most frequently mentioned well-organized aspects were multidisciplinary collaboration, referrals, contact with (patient) organizations, and the involvement of healthcare providers.

1. **What are the most important success factors you would like to maintain in collaboration with other healthcare professionals?**
   Double-click or click-and-drag the three most important success factors from the list on the right to the list on the left. Arrange the items in the left list in order of importance, placing the most important item at the top.

| - Funding for care and funding for training/education |
| --- |
| - Involvement, intention, and willingness of healthcare providers |
| - Contact with (patient) organizations |
| - Referral and transfer of care |
| - Easy, low-threshold contact between healthcare professionals |
| - Multidisciplinary coordination and consultation |

**Barriers**
In the first round of this study, collaboration between healthcare professionals in PCC care was rated on average as 5.4. 59% of participants experience challenges in collaborating with other healthcare providers. Overall, there is a perceived lack of (multidisciplinary) collaboration, with barriers to easy, low-threshold contact. There are also insufficient conditions for collaboration, such as lack of reimbursement for consultations and capacity shortages.

1. **What are the most important barriers that need to be addressed to improve your collaboration with other healthcare professionals?**
   Double-click or click-and-drag the three most important challenges from the list on the right to the list on the left. Arrange the items in the left list in order of importance, placing the most important item at the top.

| - Lack of knowledge among healthcare professionals |
| --- |
| - Lack of (multidisciplinary) collaboration between healthcare professionals |
| - Lack of reimbursement for interprofessional consultations |
| - Poor information transfer (e.g., one-way communication, lack of feedback and reporting) |
| - No low-threshold contact/short communication lines between healthcare professionals |
| - High workload and lack of capacity/time |
| - Unfamiliarity with collaboration opportunities/added value of other disciplines |
| - Differences in vision regarding treatment and guidance |

1. **To what extent do you think this barrier can be improved?** [Only for three selected answer options from question 18]

| 1  Not at all easy to improve | 2 | 3 | 4 | 5 | 6 | 7 | 8 | 9 | 10  Very easy to improve |
| --- | --- | --- | --- | --- | --- | --- | --- | --- | --- |
| ❑ | ❑ | ❑ | ❑ | ❑ | ❑ | ❑ | ❑ | ❑ | ❑ |

**Multidisciplinary collaboration**In the first questionnaire of this study, the importance of fourteen key points for multidisciplinary collaboration was assessed. The vast majority of participants (86%) considered the five points below equally important.

1. **What do you consider the most important key point for multidisciplinary collaboration?**

Only one answer is possible. We understand that these key points may vary depending on which discipline you collaborate with. Therefore, we ask you to answer this question based on the average or most common collaboration.

| - Clear communication lines and referrals |
| --- |
| - Provided in the most appropriate setting |
| - Access to care |
| - Strengthening the linkages between the work, healthcare, and social domains |
| - Expanding multidisciplinary collaboration |

**Module 4. Financial barriers in PCC care**

The following questions concern the financial barriers you experience in providing care and support to PCC patients.

Adequate financial reimbursement is frequently mentioned by participants as a prerequisite for care. In the first round of this study, 60% of participants experienced financial barriers related to the insurance package, 59% financial barriers for the patient, and 31% financial barriers for themselves as healthcare providers.

1. **What is currently the most important financial barrier for you in providing care and support to PCC patients?**

Only one answer is possible.

| - Policies, rates, and contracted care of health insurers |
| --- |
| - Limited reimbursement of care in the insurance package |
| - Funding for healthcare providers (including for multidisciplinary meetings, organization, multidisciplinary care) |
| - Lack of capacity and time in healthcare |
| - Costs and income of the patient |

1. **You indicated that limited reimbursement of care in the insurance package is the most important financial barrier for you. Which specific barriers apply to you in this regard?**

Multiple answers are possible. [Complete only if you selected “Limited reimbursement of care in the insurance package” in question 21]

| - Limited number of consultations per reimbursed treatment |
| --- |
| - Limited duration of reimbursed treatments |
| - Medical requirements for reimbursement are not met |
| - Reimbursement is not covered by basic insurance but by supplementary insurance |
| - Reimbursement of non-standard care |

**Module 5. Knowledge of PCC**

The following questions concern improving the knowledge level of PCC among healthcare professionals and the existing blind spots and knowledge gaps.

**Strategies to improve knowledge level among healthcare professionals**In the first round of this study, participants rated their own general knowledge about PCC at 7.7. Knowledge about symptoms and impact on functioning was rated highly (8.0 and 8.3, respectively), while knowledge about causes and diagnostics was rated lower (6.6 for both). The knowledge of PCC healthcare providers in general was rated at 5.7.

1. **According to you, what are the best ways to improve the knowledge level among PCC healthcare professionals in the Netherlands?**Double-click or click-and-drag the three best methods from the right-hand list to the left-hand list. Arrange the items in the left-hand list in order of importance, placing the most important item at the top.

| - Sharing advancing scientific knowledge |
| --- |
| - Implementing an integrated approach |
| - Gaining and sharing knowledge and experience |
| - More (scientific) research |
| - Raising awareness of (the severity of) PCC (e.g., professional literature, training, media) |
| - Establishing specialized PCC centers |
| - Developing, revising, and keeping multidisciplinary guidelines and recommendations up to date |
| - Improving information provision (information platform) |
| - Improving (multidisciplinary) collaboration between HCWs |
| - Education and continuing training |

**Knowledge gaps**The vast majority of participants experience blind spots or knowledge gaps regarding PCC in themselves (73%) and in other healthcare professionals (92%).

1. **Which knowledge gaps related to PCC and the care for PCC patients do you consider a priority to be addressed?**Double-click or click-and-drag the three most important knowledge gaps from the right-hand list to the left-hand list. Arrange the items in the left-hand list in order of importance, placing the most important item at the top.

| - Biomedical knowledge gaps (including underlying mechanisms) |
| --- |
| - Awareness of ongoing studies and research findings |
| - Knowledge of treatment options (including medication and follow-up care) |
| - Knowledge of exertional tolerance, PEM, and POTS |
| - Knowledge of diagnostic methods |
| - Knowledge of environmental and psychosocial factors influencing recovery (including lifestyle) |
| - Knowledge of long-term consequences, recovery, and prognosis of PCC |
| - Knowledge of PCC symptoms |
| - Knowledge of referral options and experience of other HCWs |
| - Keeping knowledge and guidelines up to date |

1. **To what extent do you think this knowledge gap can be addressed?** [Only for three selected answer options from question 24]

| 1  Not at all easy to address | 2 | 3 | 4 | 5 | 6 | 7 | 8 | 9 | 10  Very easy to address |
| --- | --- | --- | --- | --- | --- | --- | --- | --- | --- |
| ❑ | ❑ | ❑ | ❑ | ❑ | ❑ | ❑ | ❑ | ❑ | ❑ |
